# Virtual Reality-Based Screening Tool for Distance Horizontal Fusional Vergence in Orthotropic Young Subjects: A Prospective Pilot Study

**DOI:** 10.3390/life15081286

**Published:** 2025-08-13

**Authors:** Jhih-Yi Lu, Yin-Cheng Liu, Jui-Bang Lu, Ming-Han Tsai, Wen-Ling Liao, I-Ming Wang, Hui-Ju Lin, Yu-Te Huang

**Affiliations:** 1 Eye Center, China Medical University Hospital, Taichung 404327, Taiwan; jennylu0912@gmail.com; 2 Department of Information Engineering and Computer Science, Feng Chia University, Taichung 40724, Taiwanminghtsai@fcu.edu.tw (M.-H.T.); 3 Graduate Institute of Integrated Medicine, China Medical University, Taichung 406040, Taiwan; 4 Personal Medical Research Center, China Medical University Hospital, Taichung 40447, Taiwan; 5 Genetic Center, Department of Medical Research, China Medical University Hospital, Taichung 40447, Taiwan; 6 School of Chinese Medicine, China Medical University, Taichung 406040, Taiwan

**Keywords:** virtual reality, positive fusional vergence, negative fusional vergence, total vergence amplitude, screening tool

## Abstract

This prospective pilot study aimed to develop and evaluate a VR–based screening tool for assessing distance fusional vergence amplitude in healthy orthotropic young adults aged 18 to 30 years. A VR–based balloon-hitting game was used to measure hitting deviation angles and total vergence amplitudes under five conditions: control (0 prism diopter [PD]), inward image rotation for 10 and 20 PD (negative fusional vergence [NFV] 10/20 groups), and outward image rotation for 10 and 20 PD (positive fusional vergence [PFV] 10/20 groups). Of the 20 subjects recruited, one was excluded due to esotropia, leaving 19 participants (mean age: 22.2 ± 2.2 years; 13 wore glasses and 3 were female). In the control group, the mean hitting deviation was 0.65 ± 0.25 PD. The PFV 10 PD group showed similar deviation (0.67 ± 0.25 PD, *p* = 0.67), while the PFV 20 PD group had a significant increase (1.71 ± 2.0 PD, *p* = 0.04). NFV groups demonstrated greater deviations (NFV 10 PD: 3.40 ± 2.05 PD; NFV 20 PD: 9.9 ± 2.40 PD, both *p* < 0.01). Total vergence amplitudes were 8.65, 16.48, 6.60, and 10.05 PD for PFV 10, PFV 20, NFV 10, and NFV 20 PD, respectively. The VR–based tool enables standardized, efficient assessment of fusional vergence and shows promise for large-scale screening.

## 1. Introduction

Fusional vergence refers to the ability of the visual system to maintain a single, unified image by adjusting eye alignment from a naturally fused state. It is a crucial mechanism in binocular vision, enabling the eyes to remain aligned and preventing diplopia when viewing objects at different distances [1]. This process plays a fundamental role in preserving stereopsis, depth perception, and overall visual comfort in daily activities.

Fusional vergence can be classified into horizontal, vertical, and cyclofusional components [2,3,4]. Among these, horizontal fusional vergence is further divided into positive fusional vergence (PFV) and negative fusional vergence (NFV), based on the direction of eye movement. PFV involves convergence, where both eyes rotate inward to maintain fixation on near objects, while NFV involves divergence, where both eyes rotate outward to sustain alignment when viewing distant objects. An imbalance in horizontal fusional vergence can lead to symptoms such as visual fatigue, headaches, and intermittent diplopia, particularly in conditions such as convergence insufficiency/excess and divergence insufficiency/excess [5,6,7,8].

Accurate assessment of fusional vergence is essential in clinical practice for diagnosing and managing binocular vision disorders. The smooth vergence technique, performed using the Risley rotary prism in a phoropter, and the step vergence test, conducted outside the phoropter with a prism bar, are the primary assessment methods [9,10]. However, the absence of a universally accepted gold standard and the reliance on subjective patient feedback introduce variability, leading to highly heterogeneous normative values [10]. Additionally, these assessments are time-consuming, and the global shortage of pediatric ophthalmologists, exacerbated by a growing population and declining interest among ophthalmology residents, has contributed to the underdiagnosis of binocular vision disorders [11]. This issue is further exacerbated by the unequal distribution of pediatric ophthalmologists, resulting in limited access to care for patients in lower socioeconomic regions [12].

Virtual reality (VR) technology provides a controlled, immersive environment that enables precise manipulation of visual stimuli, offering a promising approach for fusional vergence assessment [13,14]. With its built-in infrared eye-tracking system and automated measurement tools, VR can be developed as a telemedicine platform or a community-based screening tool, facilitating early detection of vergence abnormalities in schools and public health programs [15]. By enhancing diagnostic accuracy and improving efficiency, VR technology may contribute to more accessible and standardized vergence assessments.

To date, no VR–based system has been developed as a screening tool for horizontal distance fusional vergence. Existing methods rely on traditional prism-based assessments, which are time-consuming and require experienced examiners. To address this gap, we launched a prospective pilot study to establish a baseline horizontal distance fusional vergence response in healthy orthotropic young subjects. In this initial phase, we aimed to test a more accessible and simplified target, serving as a foundation for future refinements. These findings will serve as a reference for future screening applications, ultimately contributing to more standardized, efficient, and effective vergence assessments.

## 2. Materials and Methods

### 2.1. Study Design

This prospective case series was conducted at China Medical University Hospital (CMUH) from August 2022 to December 2023. The study protocol was approved by the Institutional Review Board of CMUH (IRB number: CMUH111-REC1-085), and all participants provided informed consent prior to enrollment. The study adhered to the ethical principles outlined in the Declaration of Helsinki and followed internationally recognized guidelines for research and reporting standards.

### 2.2. Study Populations

Young orthotropic subjects aged 18 to 30 years were recruited from our university. Individuals with a receded near point of convergence (NPC) greater than 10 cm, assessed using fingertip testing, were excluded. Additional exclusion criteria included symptoms related to convergence insufficiency, such as headaches, diplopia, eye strain, or discomfort during near work. Participants with poor visual acuity (best-corrected VA < 20/40), difficulty maintaining attention, ptosis, or psychological conditions such as Attention Deficit Hyperactivity Disorder (ADHD) that could impact test accuracy were also excluded. To confirm orthotropia, all subjects underwent an alternate prism cover test (APCT) to exclude heterotropia. A comprehensive ophthalmological evaluation was performed by a single experienced pediatric ophthalmologist (HJL).

### 2.3. VR Equipment

In this study, we used the HTC VIVE Pro Eye, a commercially available VR system that is widely accessible worldwide. The device features dual 3.5-inch AMOLED displays with a resolution of 1440 × 1600 pixels per eye (2880 × 1600 pixels combined), a 90 Hz refresh rate, and a 110-degree horizontal field of view. We utilized VIVE controllers, which incorporate SteamVR eye-tracking technology, allowing precise gaze detection and interaction. Given its global availability and established use in professional applications, the HTC VIVE Pro Eye provides a standardized and accessible platform for VR–based research [16].

### 2.4. VR Environment and Game Design

**Figure 1:**
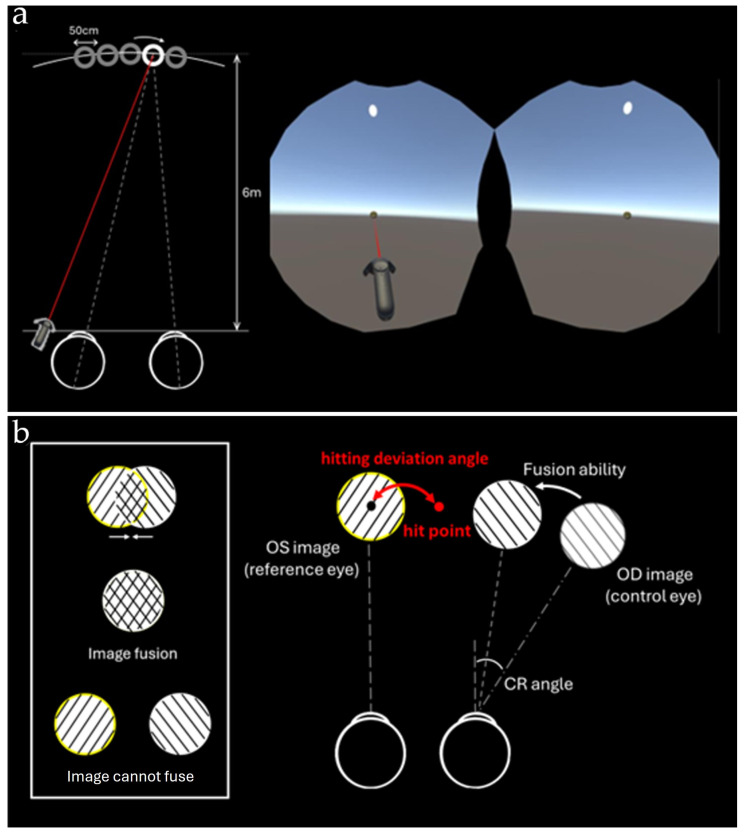
(**a**) In the VR balloon-shooting game, the participant is instructed to use a controller to hit the center of the red balloon displayed on the screen. (**b**) The angle of the image in the control eye is adjusted using the Camera Rotation method. The participant then attempts to fuse the images seen by both eyes (left side of the figure). If fusion is successful, they hit the center of the image. If fusion is unsuccessful, the participant is instructed to hit the central point of the two balloons (hit point). The angle between the hit point and the original image center is defined as the hitting deviation angle.

We developed the virtual environment using the Unity game engine, a widely compatible platform that supports various VR devices [17]. The experiment was conducted in a virtual outdoor setting, where the background consisted of a blue sky, and the ground in front of the user was gray. Within this environment, a red balloon with a diameter of 50 cm was positioned 6 m ahead of the user. The balloon’s position randomly shifted horizontally by 0 cm, 50 cm, or 100 cm from the central axis, while its height remained aligned with the user’s line of sight. Participants were required to aim at the center of the balloon and shoot using the VIVE controller, which featured a laser sight to assist with precise targeting (Figure 1a).

To simulate fusional vergence, we implemented the Camera Rotate method within the VR environment. During each trial, one eye was randomly selected as the reference eye, while the other was assigned as the control eye. The control eye’s view was modified by adjusting the VR camera’s gaze angle to induce diplopia, requiring participants to attempt fusion of the double images before shooting. If successful, they would aim at the fused balloon’s center. If fusion was not achieved, participants were instructed to shoot at the centers of both diplopic balloon images as seen by each eye (Figure 1b). After each shot, the control eye was randomly reassigned to prevent reliance on a single eye for targeting.

### 2.5. Horizontal Fusional Vergences Game Setting in Orthotropic Healthy Subjects

The VR game was designed to simulate different levels of horizontal fusional vergence in orthotropic healthy subjects. Five viewing conditions were implemented as a screening protocol: 0 PD as the control, 10 PD inward rotation (requiring the subject to diverge by 10 PD), 20 PD inward rotation (requiring the subject to diverge by 20 PD), 10 PD outward rotation (requiring the subject to converge by 10 PD), and 20 PD outward rotation (requiring the subject to converge by 20 PD). All conditions were tested in the same group of participants, and no separate control group was used; thus, demographic characteristics were identical across all conditions. The uncorrected view simulated orthotropic status.

In the inward rotation condition, subjects had to diverge their eyes to achieve fusion, engaging NFV. Conversely, in the outward rotation condition, subjects had to converge their eyes to achieve fusion, engaging PFV. The divergence exercise was tested first, followed by the convergence exercise.

During the experiment, subjects were tested under different viewing conditions in a randomized order. Each condition consisted of 40 shots, with a session duration of 1 to 2 min, depending on the subject’s ability to maintain convergence or divergence. The coordinates of the user’s hit points and the balloon’s center were recorded to calculate the deviation angle in PD along the horizontal axis. The horizontal fusional vergence value (HFV) was calculated as follows:

HFV = θ_CR − θ_HD

where θ_CR represents the camera rotation angle, and θ_HD represents the hitting deviation angle. Total vergence amplitude represents the actual amount of vergence performed by the subject and is marked as fusion ability in Figure 1b.

To assess potential VR–related discomfort, all participants completed the Simulator Sickness Questionnaire (SSQ) before and after the experiment. The SSQ is a four-point scale designed to evaluate symptoms such as nausea, oculomotor disturbances, and disorientation, ensuring that the VR environment did not negatively impact participants’ physical well-being [18].

### 2.6. Statistical Analysis

Data analysis was conducted using SPSS version 22.0 (IBM Corp., Armonk, NY, USA). Continuous variables were summarized as mean ± standard deviation (SD), while categorical data were expressed as percentages. The Shapiro–Wilk test was applied to assess normality. For comparisons involving normally distributed data, independent and paired *t*-tests were used, whereas non-normally distributed variables were analyzed using the Mann–Whitney U test or Wilcoxon signed-rank test as appropriate. The chi-square test was employed for categorical comparisons. Kendall’s coefficient of concordance was used to evaluate inter-rater reliability where applicable. All statistical analyses were two-tailed, with *p*-values ≤ 0.05 considered statistically significant.

## 3. Results

A total of 20 healthy subjects were initially screened, with one case excluded due to an incidental finding of 12 PD esotropia during the screening process. Thus, 19 subjects were included in the final analysis, with a mean age of 22.2 ± 2.2 years (range: 20–28 years), comprising 3 females and 16 males. Among all participants, 68.4% (n = 13) were tested in VR while wearing framed glasses. Baseline demographic data are presented in Table 1.

**Figure 2:**
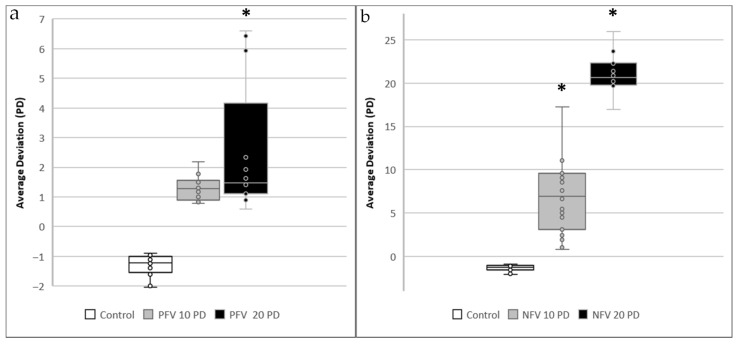
(**a**) The hitting average deviation angle in orthotropic healthy subjects under positive fusional vergence (PFV) of 10 PD and 20 PD. *: *p* < 0.05; (**b**) The hitting average deviation angle in orthotropic healthy subjects under negative fusional vergence (NFV) of 10 PD and 20 PD. *: *p* < 0.05.

In the control group, where no fusional vergence demand was applied, the mean hitting deviation was 0.65 ± 0.25 PD. In the PFV 10 PD group, where an outward image rotation of 10 PD was introduced (requiring subjects to achieve 10 PD of convergence for fusion), the mean hitting deviation remained 0.67 ± 0.25 PD, showing no significant difference compared to the control group (*p* = 0.67). However, in the PFV 20 PD group, where an outward rotation of 20 PD was applied, the mean hitting deviation increased to 1.71 ± 2.0 PD, reaching statistical significance (*p* = 0.04). Conversely, in the NFV 10 PD group, where an inward image rotation of 10 PD was introduced (requiring 10 PD of divergence to maintain fusion), the mean hitting deviation increased significantly to 3.40 ± 2.05 PD (*p* < 0.01). A similar trend was observed in the NFV 20 PD group, where an inward image rotation of 20 PD resulted in the highest recorded hitting deviation of 9.95 ± 2.40 PD (*p* < 0.01). The deviation angle measurements for all conditions are presented in Figure 2.

Total vergence amplitude, representing the actual amount of vergence performed by the subjects, varied across conditions. In the control group, the mean total vergence was 1.28 ± 0.50 PD. In the PFV 10 PD group, it increased to 8.65 ± 0.50 PD, while the PFV 20 PD group exhibited a further increase to 16.48 ± 3.99 PD. For negative fusional vergence, the NFV 10 PD group demonstrated a total vergence of 6.60 ± 2.00 PD, whereas the NFV 20 PD group reached 10.05 ± 2.34 PD. These data are presented in Table 2.

In the Simulator Sickness Questionnaire, a slight increase in scores was observed in general discomfort (1.00 to 1.10), difficulty focusing (1.05 to 1.10), difficulty concentrating (1.05 to 1.15), dizziness with eyes closed (1.00 to 1.05), and stomach awareness (1.00 to 1.05). However, none of these symptoms reached statistical significance (all > 0.05).

## 4. Discussion

In this present study, we explored the feasibility of a VR–based system for assessing distance horizontal fusional vergence in healthy orthotropic young subjects. By evaluating hitting deviation and total vergence amplitude, we aimed to provide an objective measurement of vergence ability. Our results showed that PFV at 10 PD was well maintained (*p* = 0.67), while at 20 PD, increased deviation was observed (*p* = 0.04), suggesting greater difficulty in sustaining fusion under higher convergence demands. In contrast, NFV at both 10 PD and 20 PD exhibited a more pronounced increase in deviation (both *p* < 0.01), indicating that divergence was more challenging to maintain across all tested conditions. Despite the immersive nature of VR, the system remained stable, with only slight, non-significant increases in discomfort-related symptoms reported in the SSQ. These findings support the use of VR–based screening as a viable and efficient alternative to traditional prism-based methods, providing a foundation for future applications in binocular vision assessment.

The rationale for selecting distance fusional vergence in young healthy subjects was based on several key considerations. Previous pooled analyses have shown that prism fusion ranges in children (both PFV and NFV) measured using the step vergence method exhibit high heterogeneity at both near and distance (I^2^ = 51–93%) compared to adults (I^2^ = 0–36%) [1]. This variability suggests that fusional vergence in young adults may provide more reliable and comparable results with prior studies. Additionally, because our study did not assess the AC/A ratio, and Sharma et al. reported high variability in base-in vergence findings at near [19], we chose fusional vergence at distance as the focus of our pilot study. Furthermore, Álvarez et al. found that age has a significant negative correlation with fusional vergence recovery, with an approximate reduction of 0.05 PD/year for base-in vergence and 0.07 PD/year for base-out vergence [9]. These findings align with other studies reporting a decline in vergence abilities among elderly subjects [20], so we aimed to target our subjects toward younger populations.

The selection of 10 PD and 20 PD as screening values aligns with previous studies. In participants aged 18 to 70 years, tested using the step vergence method, the distance PFV break point has been reported to range from 16 to 25 PD, with a standard deviation of 1.5 to 4.3 PD [19,21,22,23]. For NFV, the break point typically falls between 5.6 and 9.7 PD, with a standard deviation of 4.7 to 10.2 PD [22,23,24]. Similarly, when fusional vergence is measured using the smooth vergence method (Risley rotary prism), the distance PFV break point is reported to range from 10.9 to 24.7 PD, with a standard deviation of 4.6 to 9.0 PD [9,24,25]. The NFV break point under this method typically falls between 7.4 and 13.0 PD, with a standard deviation of 3.4 to 4.6 PD [25,26]. Our results from the 20 PD group fall within the range of previous literature, with PFV at 16.48 ± 3.99 PD and NFV at 9.95 ± 2.40 PD, further validating the reliability of our VR–based measurement platform.

Several factors required consideration in this study. First, ocular dominance has been suggested as a factor influencing fusional reserves, but findings remain inconsistent [27,28]. To account for potential differences in measurements between the dominant and non-dominant eye, we randomly assigned either eye as the reference in each session. Second, examiner encouragement has been linked to better convergence performance but has little impact on divergence [24,29]. In this study, we aimed to actively encourage participants to achieve the best possible fusion, maximizing their fusional potential. To minimize learning curve influences, we selected young adult participants who were more familiar with the VR–based hitting game used in this study. Additionally, all subjects completed a pretest session to become accustomed to the game environment. Lastly, the order of assessment has been shown to affect fusional vergence outcomes, with studies reporting that divergence amplitudes decrease when tested after convergence, likely due to vergence adaptation or fusional after-effects [24,29]. To prevent this issue, we tested divergence before convergence.

Monier et al. conducted a VR–based vergence study in 50 psychology undergraduates and found that vergence scores for convergence and divergence in VR were very similar to prism-based results, indicating effective stimulation of 3D visual conditions. They also showed that controlled virtual environments provided better conditions for measuring vergence movements and that VR prevented underestimation of divergence in participants with high convergence abilities [30]. Our study, focusing on the feasibility of VR as a clinical screening tool with rapid measurement and standardization, further supports the potential and applicability of VR in this field.

VR–based fusional vergence assessment differs fundamentally from traditional techniques such as prism bars or phoropters. Traditional methods are widely available, low-cost, and clinically familiar but rely heavily on examiner expertise and subjective patient feedback, leading to variability in results. VR–based methods provide standardized, automated measurements with immersive control of visual stimuli, enabling more precise manipulation of testing conditions. Although current application in routine clinical practice may be limited by equipment cost and technical requirements, VR technology holds considerable future potential for broader use, including orthoptic exercise programs, community screening, and telemedicine applications.

The strength of the present study lies in being the first VR–based screening tool for fusional vergence, offering an innovative and efficient approach to vergence assessment. This VR–based system provides a standardized and automated screening process, reducing reliance on examiner expertise. By focusing on NFV and PFV at 10 PD and 20 PD, the test remains both time-efficient and clinically practical, making it a feasible option for large-scale screening. Furthermore, our pilot study aligns well with results obtained from traditional methods, further reinforcing the reliability of this VR platform. Additionally, VR–based assessments have the potential to enhance compliance in children, who are often considered a high-variability group in traditional vergence testing.

Despite its strengths, this study has several limitations. First, it serves as a screening tool rather than a direct measurement of fusional vergence break and recovery points. We speculate that PFV values may increase if larger rotations were provided, warranting future studies to determine the full vergence range. Another limitation involves potential sources of error within the VR system, which may require further calibration to improve accuracy. Another limitation is the lack of detailed refractive data. Although most participants wore their daily spectacles and met our BCVA criteria, residual uncorrected refractive errors could still influence fusional vergence measurements. Future studies should incorporate full refraction data to address this issue. Another limitation is the potential influence of interpupillary distance (IPD) alignment on measurement accuracy and participant comfort. Although IPD was measured before the test, small residual misalignments could still contribute to visual discomfort and data variability. Prior research has shown that even a small IPD mismatch can significantly increase symptoms of dizziness, headache, and eye discomfort, underscoring the importance of precise calibration for reliable results [31]. Additionally, the relatively small sample size and the lack of refraction data and AC/A ratio measurements highlight the need for larger-scale validation studies to confirm the generalizability of our findings across other age groups and clinical conditions.

In conclusion, this prospective pilot study introduces the first VR–based screening tool for fusional vergence. By assessing NFV and PFV at 10 PD and 20 PD, this system offers an efficient and standardized alternative to traditional assessment methods, making it a promising option for large-scale screening applications. Future research should explore total vergence amplitude, conduct further validation studies, and assess the broader potential of VR–based binocular vision screening, telemedicine applications, and orthoptic therapy.

## Figures and Tables

**Table 1:** Demographic and clinical characteristics of the subjects.

Age in Years, Mean (Range)	22.2 ± 2.2, (20–28)
Female n (%)	3 (15.8%)
Measurements obtained in glasses n (%)	13 (68.4%)

The mean ± standard deviation was used for continuous variables, and n (%) for nominal variables.

**Table 2:** Deviation angle and total vergence at different fusional vergence levels.

Target Vergence by Distance	Total Vergence (PD)	Average Deviation (PD)	*p* Value
Control, 0 PD	1.28 ± 0.50	0.65 ± 0.25	Control
PFV 10 PD	8.65 ± 0.50	0.67 ± 0.25	0.67
PFV 20 PD	16.48 ± 3.99	1.71 ± 2.01	0.04 *
NFV 10 PD	6.60 ± 2.00	3.40 ± 2.05	<0.01 *
NFV 20 PD	10.05 ± 2.34	9.95 ± 2.40	<0.01 *

PD: prism diopter, PFV: positive fusional vergence, NFV: negative fusional vergence, *p*-values indicate statistical comparisons of average deviation in each subgroup relative to the control condition; * = *p* < 0.05; the mean ± standard deviation was used for continuous variables.

## Data Availability

The original contributions presented in this study are included in the article. Further inquiries can be directed to the corresponding authors.

## Abbreviations

The following abbreviations are used in this manuscript:

PD	prism diopter
NFV	negative fusional vergence
PFV	positive fusional vergence
VR	virtual reality
NPC	near point of convergence
ADHD	Attention Deficit Hyperactivity Disorder
APCT	alternate prism cover test
SSQ	Simulator Sickness Questionnaire
SD	standard deviation
